# Prediction of the number of positive axillary lymph nodes according to sentinel lymph node involvement and biological subtypes in patients receiving neoadjuvant chemotherapy

**DOI:** 10.1186/s12893-024-02500-5

**Published:** 2024-07-19

**Authors:** Latif Yilmaz, Tulay Kus, Alper Aytekin, Gokmen Aktas, Evren Uzun, Gokturk Maralcan

**Affiliations:** 1 Department of General Surgery, School of Medicine, Gaziantep University, Gaziantep, TR-27310 Turkey; 2 Department of Medical Oncology, School of Medicine, Gaziantep University, Gaziantep, TR-27310 Turkey; 3 Department of Medical Oncology, Gokmen Aktas, Assoc Prof. Gaziantep Medical Point Hospital, Gaziantep, TR-27584 Turkey; 4 Department of Pathology, School of Medicine, Gaziantep University, Gaziantep, TR-27310 Turkey; 5 Department of General Surgery, School of Medicine, Sanko University, Gaziantep, TR-27090 Turkey

**Keywords:** Prediction, Axillary lymph node dissection, Stages, Sentinel lymph node biopsy, Luminal subtypes, Neoadjuvant chemotherapy

## Abstract

**Background:**

Sentinel lymph node biopsy (SLNB) has replaced axillary lymph node dissection (ALND) for assessing axillary lymph node status in clinically node-negative breast cancer patients. However, the approach to axillary surgery after neoadjuvant treatment is still controversial. In the present study, our objective was to predict the pathological nodal stage based on SLNB results and the clinicopathological characteristics of patients who initially presented with clinical N1 positivity but whose disease status was converted to clinical N0 after neoadjuvant chemotherapy (NAC).

**Materials and methods:**

After NAC, 150 clinically node-negative patients were included. The relationships between clinicopathologic parameters and the number of positive lymph nodes in SLNBs and ALNDs were assessed through binary/multivariate logistic regression analysis.

**Results:**

Among 150 patients, 78 patients had negative SLNBs, and 72 patients had positive SLNBs. According to the ALND data of 21 patients with SLNB1+, there was no additional node involvement (80.8%), 1–2 lymph nodes were positive in 5 patients (19.2%), and no patient had ≥ 3 lymph nodes involved. Following the detection of SLNB1 + positivity, the rate of negative non-sentinel nodes were 75% in the luminal A/B subgroup, 100% in the HER-2-positive subgroup, and 100% in the triple-negative subgroup. Patients with a lower T stage (T1-3 vs. T4), fewer than 4 clinical nodes before NAC (< 4 vs. ≥4), and a decreased postoperative Ki-67 index (< 10% vs. stable/increase) were included. According to both univariate and multivariate analyses, being in the triple-negative or HER2-positive subgroup, compared to the luminal A/B subgroup (luminal A/B vs. HER2-positive/triple-negative), was found to be predictive of complete lymph node response.

**Conclusion:**

The number of SLNB-positive nodes, tumor-related parameters, and response to treatment may predict no additional nodes to be positive at ALND.

**Supplementary Information:**

The online version contains supplementary material available at 10.1186/s12893-024-02500-5.

## Introduction

The optimal treatment for regional lymph nodes in clinically node-negative patients after neoadjuvant therapy is currently unclear, and treatment algorithms are needed for fewer surgeries in favor of more radiotherapy (RT). Sentinel lymph node biopsy (SLNB) has replaced axillary lymph node dissection (ALND) as the preferred method for assessing axillary lymph node status in clinically node-negative breast cancer patients who undergo upfront surgery due to its high negative predictive value [1]. Two large randomized phase III trials have shown that axillary lymph node dissection can be omitted in patients with limited disease in the sentinel node or nodes who are treated with whole-breast irradiation and adjuvant systemic treatment without compromising locoregional control or overall survival (OS) [2, 3]. The 10-year follow-up of ACOSOG Z0011, a Phase III trial, showed that SLNB alone was as effective as ALND for overall survival (OS) in patients with 1, 2, or 3 positive sentinel lymph node metastases [2]. Another phase 3 noninferiority trial, AMAROS, also demonstrated that the ten-year overall survival (OS) rate was 84.6% in the ALND group and 81.4% in the SLNB alone group (HR, 1.17; 95% CI, 0.89 to 1.52; *P* = 0.26). Additionally, the 10-year disease-free survival rate was 75.0% in the ALND group and 70.1% in the SLNB alone group (HR, 1.19; 95% CI, 0.97 to 1.46; *P* = 0.11) [3]. Therefore, the current guidelines recommend that ALND be omitted in patients with 1 or 2 positive SLNBs who underwent upfront surgery [4]. The most significant feature of these two practice-changing studies was that the patients included had T1-2 tumors, with the majority of tumors being less than 2 cm in size. Additionally, the majority of patients were classified into endocrine-positive and HER2-negative pathological subgroups. Therefore, the applicability of this approach in larger tumors, HER2-positive or triple-negative patients, and patients treated with neoadjuvant therapy (NAT) remains unclear for clinicians [2, 3].

Currently, where systemic treatments have become notably potent, neoadjuvant therapy (NAT) is now recommended for the majority of patients. Moreover, the presence of residual disease after NAT necessitates further systemic adjuvant therapy. Consequently, there is a need to establish clear guidelines for managing the axilla after neoadjuvant therapy (NAT). The presence of remaining lymph nodes after NAT may indicate a potential chemotherapy-resistant clone. Surgical dissection could provide benefits in terms of controlling local recurrence and overall survival, especially for patients who have upfront surgery. Therefore, long-term survival results from studies in which ALND is omitted in patients with positive sentinel lymph nodes following NAT are needed [5]. Furthermore, ALND plays a crucial role in achieving accurate nodal staging. It is well established that prognosis worsens in correlation with the presence and increase in the number of residual lymph nodes [6]. Moreover, in determining the need for additional adjuvant systemic therapy, while the presence of residual disease suffices for the HER-2-positive and triple-negative breast cancer subgroups, accurate node staging becomes essential for considering the inclusion of chemotherapy or CDK4/6 inhibitors alongside endocrine therapy in the hormone-positive, HER-2-negative patient subgroup [7, 8]. ALND in the presence of positive SLNBs is crucial for establishing an accurate prognosis and guiding treatment decisions after NAT. Nevertheless, preliminary results from ongoing studies suggest that the future may lead to reduced surgical interventions not only in clinically node-negative patients but also in clinically node-positive patients after NAT. In the present study, our objective was to predict the number of lymph node involvement at ALND based on the SLNB results and the clinicopathological characteristics of patients who initially presented with clinical N1 positivity but were converted to clinical N0 status after NAT.

## Materials and methods

### Patient selection, surgery and systemic treatments

Breast cancer patients who were under the care of the Department of General Surgery at Gaziantep University and Sanko University were retrospectively reviewed from January 2018 to August 2023. Patients were eligible for inclusion if they presented with clinically staged T1-4 N1M0 disease, as per the American Joint Committee on Cancer TNM staging system, and subsequently showed a return to clinically negative lymph nodes following neoadjuvant chemotherapy (NAC). Patients with matted/fixed lymph node (clinical N2) or level III lymph node involvement and with inflammatory breast cancer, patients who did not complete NAC, or those with missing data on clinical or pathologic stage and receptors were excluded.

Clinical N1 disease was defined by the presence of movable ipsilateral level I-II axillary lymph nodes as detected through physical examination or by biopsy-confirmed metastases in level I-II axillary nodes or by the presence of features highly suggestive of malignancy based on imaging. All patients with clinically negative axilla underwent breast surgery with the intention of curative treatment, and nodal staging was performed through sentinel lymph node biopsy (SLNB) after completing NAC. After the completion of NAC, axillary lymph node dissection (ALND) was performed for patients with clinically positive axilla.

Lymphatic mapping for SLNBs was achieved using a single tracer, either a radioactive pharmaceutical agent labeled with Tc-99 m, a blue dye, or both. The radioactive agent and blue dye were injected intradermally in the subareolar space. Radioactively labeled lymph nodes were detected intraoperatively using a gamma probe, while lymph nodes marked with blue dye and radioactive material were considered sentinel lymph nodes. Targeted axillary dissection was also performed for patients in whom clips were placed on the axillary lymph node before NAC. Blue-dyed lymph nodes (sentinel lymph nodes), if present, targeted lymph nodes, and other lymph nodes suspected to be pathological during dissection (nonsentinel lymph nodes) were removed and sent for frozen examination and postoperative serial H and E sectioning. At least 3 lymph nodes were sent for frozen examination, and postoperative serial H and E sections were obtained from each patient. The assessment of SLNB positivity was based on the total pathological positivity of the sentinel, targeted, and nonsentinel lymph nodes. If there were no metastases in these removed lymph nodes, SLNB was considered negative, and ALND was not performed. Patients who had metastases in at least 1 lymph node were considered SLNB-positive and then underwent ALND. The number of positive findings on SLNB was noted for each patient and categorized into 0–3 (0 = 0; 1 = 1; 2 = 2; 3 = 3 or more positive lymph nodes) for analysis. The ALND result was determined based on the pathological evaluation of the number of positive lymph nodes and was categorized as “node negative”, “node 1–2 positive”, or “≥3 node positive”.

All patients were treated with neoadjuvant anthracycline with cyclophosphamide every 2 or 3 weeks for 4 cycles and then with weekly paclitaxel (or three weekly docetaxel) with or without carboplatin ± Herceptin/pertuzumab (in the case of Her-2-positive disease) for 12 weeks.

All patients were screened using 18 F-FDG-PET/CT, ultrasonography (USG), and in some cases, mammography and breast MRI at the time of diagnosis. Based on the tumor board decision, patients without high suspicion of axillary lymph node involvement on imaging underwent lymph node biopsy before NAC. Patients who were clinically and radiologically determined to have node-negative disease by 18FDG-PET/CT and USG following the completion of neoadjuvant chemotherapy were included in the study. The patients were categorized into three pathological subgroups based on their immunohistochemistry (IHC) and fluorescence in situ hybridization (FISH) results: luminal A-B disease: Patients were considered to have luminal A-B disease if they were estrogen receptor (ER) and/or progesterone receptor (PR) positive and HER2 negative according to IHC and FISH. The luminal B subtype was defined as ER-positive and HER2-negative, with either a high Ki67 or low PgR. HER2-positive disease Patients were categorized as HER2 positive if they were HER2 +++ positive according to IHC or + + positive according to IHC and were FISH positive, regardless of their ER or PR status. Triple-negative disease: Patients were classified as having triple-negative disease if all receptor staining (ER, PR, or HER2) was negative. Ki-67 levels were assessed at the time of diagnosis and after neoadjuvant therapy. The patients were divided into two groups: those whose Ki-67 levels decreased to less than 10% and those whose Ki-67 levels did not decrease below 10% or remain stable. The following parameters were recorded: patient age, menopausal status, pathological subtype (invasive ductal carcinoma vs. invasive lobular carcinoma), preoperative number of pathological lymph nodes (< 3 lymph nodes vs. ≥ 3 lymph nodes), T stage (T4 vs. T1-3), presence or absence of multicentric disease, localization of the primary breast tumor (left/right; upper medial or lateral; lower medial or lateral quadrant), preoperative Ki-67 (as a continuous variable) and postoperative Ki-67 (decrease in < 10% vs. none (≥ 10 Ki-67 or stable or increase).

This two-center retrospective study included 150 breast cancer patients who had undergone surgery. This study was approved by the Gaziantep University Faculty of Medicine Ethics Committee (no: 2023/228) and conducted in compliance with ethical principles according to the Declaration of Helsinki.

### Statistical analysis

The descriptive statistics of the data included the mean and standard deviation for the numerical variables and the frequency and percentage for the categorical variables. The axillary nodal disease burden was stratified by the presence of SLNB and/or ALND pathological results. Binary logistic regression analysis was used to assess the associations between N0 and clinicopathological parameters, and multivariable logistic regression was subsequently performed for parameters < 0.10. Analyses were carried out with the help of the SPSS 22.0 program. A significance level of *p* < 0.05 was chosen.

## Results

**Fig. 1:**
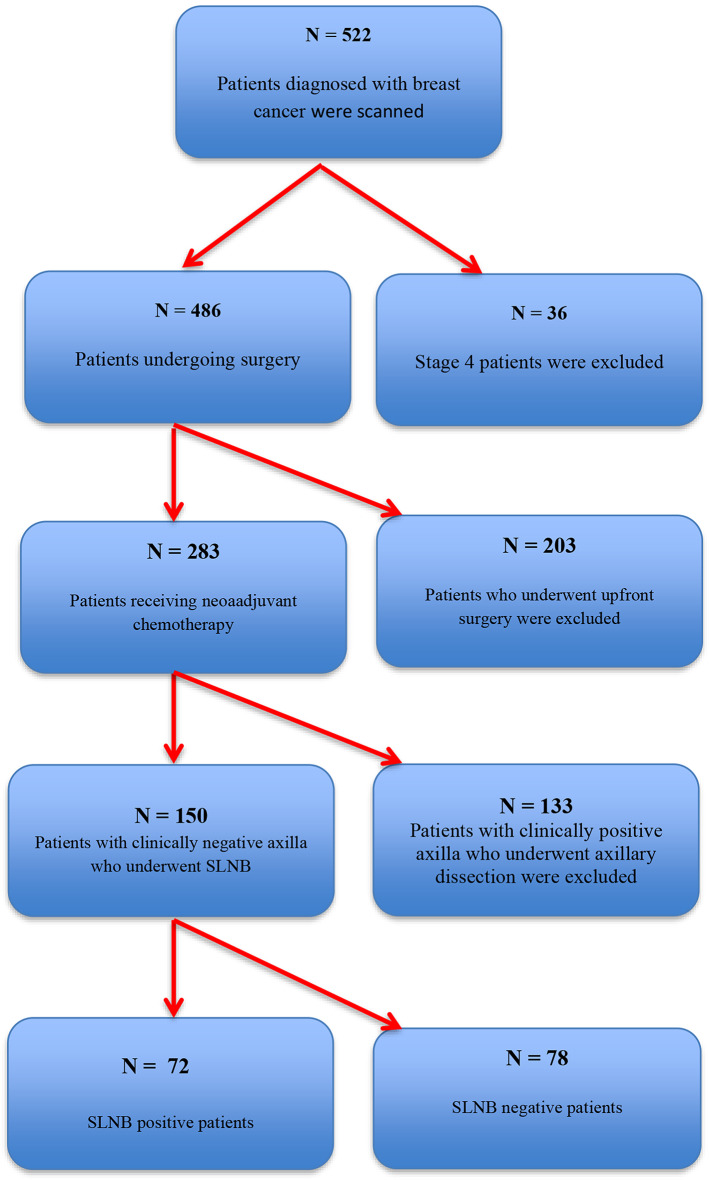
Flow chart of the study. N = number of patient

The screening and inclusion criteria for patients are shown in Fig. 1, the consort diagram. Initially, a total of 150 patients with invasive breast cancer who were clinically N1 positive but later became clinically negative after neoadjuvant therapy and subsequently underwent curative surgery were included in the study. The median age was 47 (range: 25–81) years. Of the patients, 62.7% were premenopausal, and the remaining were postmenopausal. The majority of patients had clinical T1-2 tumors (82.0%) and were clinically positive in 1 to 2 lymph nodes (82.0%). In total, 92.7% of patients had ductal histology. The tumor characteristics and other pathological features are summarized in Table 1.

**Table 1:** Clinicopathological characteristics of the patients

Parameters	*N* (%)
**Age**,** years**,** median (range)**	47 (25–81)
Menopausal status Premenopausal Postmenopausal	94 (62.7) 56 (37.3)
Pre-NAC tumor size, mm, mean (± SD)	27.21 ± 1.69
T stage 1 2 3 4	45 (30.0) 78 (52.0) 5 (3.3) 22 (14.7)
Pre-NAC lymph node, number <3 ≥3	123 (82.0) 27 (18.0)
Localization Left Right Bilateral	76 (50.7) 73 (48.7) 1 (0.7)
Tumor location Upper medial quadrant Upper lateral quadrant Lower medial quadrant Lower lateral quadrant Retroareolar	27 (18.1) 70 (47.0) 10 (6.7) 22 (14.8) 20 (13.4)
Multicentric Yes No	1 (0.6) 149 (99.4)
Histology Ductal Lobular	139 (92.7) 11 (7.3)
Post-operative Ki-67 Decrease in < 10% Stable/ increase/ or ≥ 10% Missing	76 (50.7) 53 (35.3) 21 (14.0)
Luminal sub groups Luminal A Luminal B Luminal-her-2 positive Her-2 positive Triple negative	29 (19.3) 51 (34.0) 23 (15.3) 20 (13.3) 27 (18.0)

NAC: Neoadjuvant chemotherapy

### Prediction of the number of positive axillary lymph nodes at ALND based on SLNB positivity

We did not perform ALND on 78 patients who were considered SLNB-negative when no positivity was detected in the blue-dyed (sentinel), targeted, or nonsentinel lymph nodes. However, ALND was conducted on 72 patients who were considered SLNB-positive when at least one positive result was detected in the blue-dyed (sentinel), targeted, or nonsentinel lymph nodes. The numbers of positive axillary lymph nodes in patients who underwent ALND based on SLNB 1+, 2+, and 3 + positivity are presented in Table 2. Among the 34 of 72 patients (47.2%) who underwent axillary dissection due to SLNB positivity, no lymph node metastasis was detected in the axillary dissection material. According to the ALND data of 21 patients with SLNB1+, there was no additional node involvement (80.8%), 1–2 lymph nodes were positive in 5 patients (19.2%), and no patient had ≥ 3 lymph nodes involved (Table 2). The percentages of negative lymph nodes at ALND, except for SLNBs, were 21 (80%), 10 (41.7%), and 3 (13.6%) for the SLNB1+, SLNB2 + and SLNB3 + patients, respectively.

**Table 2:** Additional positive axillary lymph node involvement according to the number of positive sentinel lymph nodes

*N* = 72	Node negative *N* (%)	1 to 2 Node Positive *N* (%)	≥ 3 Node Positive *N* (%)
SLNB 1+ *N* = 26	21 (80.8)	5 (19.2)	0
SLNB 2+ *N* = 24	10 (41.7)	7 (29.2)	7 (29.2)
SLNB 3+ *N* = 22	3 (13.6)	8 (36.4)	11 (51.0)

SLNB: sentinel lymph node biopsy; * Number of axillary lymph node metastases except for the number of positive sentinel lymph node biopsy

### Prediction of the number of nodal metastases based on SLNB positivity in patients who underwent ALND, with a focus on the luminal subtypes

According to these findings, in the luminal A/B, HER-2-positive, and triple-negative subtypes, the percentages of patients with no SLNB positivity after NAC were 32.5%, 69%, and 82.1%, respectively. Following the detection of SLNB1 + positivity, the percentages of patients with a complete lymph node response were 75%, 100%, and 100%, respectively (Table 2). Thus, the percentage of SLNB-negative patients was greater in the HER-2-positive or triple-negative patient group. Furthermore, in the case of SLNB 1 + positivity, it was observed that all patients in the HER-2-positive and triple-negative patient groups had no additional nodes positive at ALND. Her2 + and triple-negative disease were more likely to be associated with no additional nodes being positive at ALND, even in the setting of SLN1+. In the luminal A/B subgroup, among patients with a 1 + SLNB, 25% were classified as having 1–2 positive lymph nodes at ALND, and no patients had ≥ 3 positive lymph nodes. Among these patients, 4 had 1 positive lymph node, and 1 had 2 positive lymph nodes.

### Clinicopathological factors predicting SLNB-negative patients and all ALND-negative patients

We analyzed the parameters predicting patients who were SLNB-negative and those who underwent ALND and had no observed lymph node metastasis. Based on this analysis, there were no relationships between pN0 and age (< 45 vs. ≥45), menopausal status (premenopausal vs. postmenopausal), tumor location (right breast vs. left breast), tumor location (upper-inner, upper-outer, lower-inner, lower-outer, retroareolar), or baseline Ki-67 value (continuous). According to our univariate analysis, compared with ILC, IDC seemed to be a better predictor of lymph node negativity. However, its effectiveness was not observed in multivariate analysis. This could be related to the fact that the ILC subtype is often understaged using routine conventional imaging methods. A lower T stage (T1-3 vs. T4), fewer than 4 clinical nodes before NAC (< 4 vs. ≥4), a postoperative Ki-67 decrease (< 10% vs. stable/increase), and being in the triple-negative or HER2-positive subgroup compared to luminal A/B (luminal A/B vs. HER2-positive/triple-negative) were found to be predictive parameters for negative lymph nodes in both univariate and multivariate analyses (Tables 3 and 4).

**Table 3:** Univariable analysis of predictors of SLNB negative and/or, ALND negative lymph nodes among clinically T1-4 N1 patients who returned clinically N0 after neoadjuvant therapy (*n* = 150)

Parameters	Odds Ratio; 95%Cl	*P* value
Age ≤45 >45	1.35 (0.65–2.84) 1 (ref)	0.42
T stage T1-3 T4	7.58 (2.86–20.11) 1 (ref)	***< 0.001***
Pre-NAC lymph node, number <4 ≥4	10.3 (4.05–26.2) 1 (ref)	***< 0.001***
Localization Left Right	1.31 (0.62–2.77)	0.48
Tumor localization Upper medial quadrant Upper lateral quadrant Lower medial quadrant Lower lateral quadrant Retroareolar	**-**	0.89
Histology Ductal Lobular	4.01 (1.1–14.1) 1 (ref)	***0.029***
**Ki-67 prior NAC** **(continuous variable)**	0.986 (0.968–1.003)	0.112
Post-operative Ki-67 Decrease in < 10% Stable/ increase/ or ≥ 10%	7.56 (2.93–19.5) 1 (ref)	***< 0.001***
Luminal sub groups Luminal A/B Her-2 positive or Triple negative	1 (ref) 4.65 (1.96–11.1)	***< 0.001***

NAC: Neoadjuvant chemotherapy

**Table 4:** Multivariable analysis of predictors of SLNB negative and/or, ALND negative lymph nodes among clinically T1-4 N1 patients who returned clinically N0 after neoadjuvant therapy (n = 150)

Parameters	Odds Ratio; 95%Cl	P value
T stage T1-3 T4	8.75 (2.34–32.66)	***0.001***
Pre-NAC lymph node, number <4 ≥4	4.92 (1.53–15.86)	***0.008***
Histology Ductal Lobular	1.82 (0.31–10.87)	0.514
Post-operative Ki-67 Decrease in < 10% Stable/ increase/ or ≥ 10%	5.11 (1.68–15.53)	***0.004***
Luminal sub groups Luminal A/B Her-2 positive or Triple negative	5,85 (1,59 − 21,39)	***0.008***

NAC: Neoadjuvant chemotherapy

## Discussion

Axillary dissection should be avoided in current publications because of its significant complications and high mortality rates. The necessity of ALND is determined by SLNB [9]. Some studies examine whether patients with clinically node-positive breast cancer, who were converted to clinically node-negative after neoadjuvant therapy, can safely undergo SLNB [10, 11]. Some studies investigate if patients receiving NAT who had SLNB-negative can avoid unnecessary surgery because ALND is not performed [12]. Guidelines still recommend ALND in breast cancer patients receiving NAT if SLNB-positive results [13]. The aim of our study was to examine whether ALND could be omitted for breast cancer patients with SLNB-positive who were clinical axilla-negative patients after NAT.

Understanding the presence of lymph node metastasis and the true nodal stage at the regional microscopic level provides crucial information about disease prognosis and treatment decisions. It is important to note that while more aggressive surgery may not necessarily contribute to improved survival, a more intensive systemic treatment approach is often needed to improve the prognosis [14, 15]. The de-escalation of axillary surgery in patients with clinically node-positive breast cancer is currently limited to the neoadjuvant setting, contingent upon the demonstration of pathological complete response through sentinel lymph node evaluation, as supported by long-term follow-up data from database-based studies [16, 17]. In our study, among the 150 patients initially diagnosed as clinically node-positive but subsequently achieving clinical node negativity after neoadjuvant therapy, ALND was omitted in 78 patients where no positivity was observed following SLNB or removal of marked lymph nodes. On the other hand, 72 patients with SLNB positivity, whether involving sentinel or marked nonsentinel lymph nodes, underwent ALND independent of the number of positive lymph nodes. In 34 of the 72 patients who underwent ALND (47.2%), no metastases were found in the lymph nodes removed during ALND, except for those identified through sentinel lymph node biopsy (SLNB). This finding suggested that, in fact, surgery was performed in 47.2% of patients who needn’t underwent ALND. Therefore, in our study, we analyzed the clinicopathological characteristics of patients to predict those who were either SLNB-negative or SLNB-positive but had no additional lymph nodes at ALND. According to these findings, the percentages of patients who were SLNB-negative after NAC in the luminal A/B, HER-2-positive, and triple-negative subtypes were 32.5%, 69%, and 82.1%, respectively. Following the detection of SLNB1 + positivity, the percentages of patients with no additional lymph nodes at ALND were 75%, 100%, and 100%, respectively. Therefore, in the HER-2-positive or triple-negative pathological subgroups, there was not only a high chance of achieving a complete lymph node response, as described in the literature but also a high likelihood of no lymph node involvement being observed after ALND in the presence of SLNB 1 + disease. Moreover, while SLNB positivity provides guidance for additional systemic treatment management, ALND seems to be an unnecessary surgery for patients with SLNBs 1+, particularly in HER2 + or triple-negative patients. In patients with 2 + SLNB, the percentage of patients with no additional lymph nodes at the ALND was 41.7% for all pathological subgroups and 13.6% for 3 + SLNB. According to our study, we demonstrated that the number of involved sentinel lymph nodes can predict true nodal staging, especially when considering luminal pathological subgroups.

In a database study, omission of ALND was shown to be associated with inferior survival in breast cancer patients with residual N1 nodal disease following neoadjuvant chemotherapy [18, 19]. In that study, patients were categorized into those who received SLNB (defined as the removal of ≤ 4 lymph nodes) and those who received radiotherapy or ALND and radiotherapy by propensity score matching analyses. Moreover, SLNB was shown to be associated with significantly worse OS in multivariate analyses (HR: 1.7; 95% CI 1.3–2.2; *P* < 0.001), with estimated 5-year OS rates of 71% and 77% in the SLNB group and ALND group, respectively (*P* = 0.01). Accordingly, a lower tumor grade, T stage, receipt of endocrine therapy and residual disease in a single lymph node were associated with improved survival. An exploratory subgroup analysis was performed on patients in the hormone-positive HER-2-negative subgroup, which showed that SLNB was comparable to ALND in patients with single metastatic lymph node involvement. The estimated 5-year OS was 85% for SLNB and 82% for ALND (HR = 1.03; 95% CI = 0.59–1.8; *P* = 0.91). These results show that, similar to studies on SLNB in upfront surgery, the number of residual positive SLNBs after NAC is crucial. Not all patients with residual positivity were considered to be in the same category for ALND indication. In our study, considering the results of the aforementioned database-based study, it appears that the SLNB 1 + luminal A/B subgroup with ALND (y)PN1 (due to the finding of at least one positive SLN) (75%) underwent unnecessary surgery, and patients with ALND pN1 (25%; 4 patients had 1 lymph node; 1 patient had 2 positive lymph nodes) would likely yield similar survival outcomes as those without ALND. Moreover, as mentioned above, in the HER2-positive and triple-negative subgroups, all SLNB 1 + patients were nodal negative after ALND, regardless of the initial T stage and number of nodal involvement. Considering this, although waiting for the results of the Phase III study analyzing the omission of ALND after NAC might be more appropriate [5], omitting ALND in the SLNB 1 + patient group can be considered in both hormone-positive and HER2-positive and triple-negative patient subgroups.

A pooled analysis demonstrated that the residual cancer burden (RCB) score, calculated based on primary tumor dimensions, tumor bed cellularity, and axillary nodal burden, was prognostic for each breast cancer subtype following neoadjuvant therapy [6]. Furthermore, RCB was found to be a prognostic factor independent of pretreatment clinicopathological features and irrespective of hormone receptor and HER2 subtypes. Therefore, the specific number of lymph nodes involved in the residual tissue is important because it plays a crucial role in determining patient prognosis. In our study, as previously mentioned, we observed that all SLNB 1 + patients in the luminal A/B subgroup had either ALND (y)pN1 or pN1. Among patients with 2SLNB, 29.2% had pN1 (1–3 positive nodes), while 29.2% had pN2-3 (≥ 4 positive nodes) following ALND. Among those with SLNBs 3+, 36.4% had pN1, and 50.1% had N2-3. Therefore, as the percentage of SLNBs increases, the nodal stage also increases, leading to a worse prognosis. In such cases, if ALND is not performed, for SLNB-positive, hormone-positive, HER2-negative patients who receive neoadjuvant endocrine therapy, genomic recurrence score tests such as Oncotype can be considered to guide the omission of chemotherapy in the SLNB 1 + patient subgroup. However, for patients with SLNBs ≥ 2, given the high risk of potential N2-3 staging, these patients may be considered definite candidates for chemotherapy if ALND is not performed.

When the parameters predicting patients with pathological pN0 were analyzed, it was observed that the HER2-positive and triple-negative subgroups were 5.9 times more advantaged in multivariate analysis than was the hormone-positive HER2-negative luminal A-B subgroup (OR: 5.85; 95% CI: 1.59–21.4; *p* = 0.008). In addition, both univariate and multivariate analyses revealed that among the clinical and pathological factors, having T1-3 disease compared to T4 disease, having 3 or fewer lymph nodes before NAT compared to having more than 3, and having a post-NAC Ki-67 index less than 10% compared to remaining above 10% were predictive parameters for nodal-negative disease. Although being diagnosed with IDC was predictive according to univariate analysis, it lost its predictive value in multivariate analysis when compared to that of ILC. This phenomenon might be associated with the understaging of ILCs using conventional imaging methods. In fact, it is well known that the chances of achieving nodal-negative disease after NAT decrease as the T and N stages increase, which aligns with the literature [14, 20]. However, in our study, for the first time, we demonstrated that patients with Ki-67 levels decreasing below 10% after NAT had a high chance of achieving nodal negativity (OR = 5.11; 95% CI = 1.68–15.5; *P* = 0.004 in multivariate analysis). The WSG-ADAPT HR+/HER2- trial, which explored the omission of chemotherapy in hormone-positive and HER2-negative patients after NAT, showed that a post-NAT Ki-67 level less than 10% is a favorable prognostic biomarker [21].

The American College of Surgeons Oncology Group Z1071 Phase II trial reported a 12.6% false-negative rate (FNR) for SLNB after neoadjuvant chemotherapy in clinical N1 (cN1) breast cancer [22]. In their multivariable analyses, including age, body mass index, T stage, chemotherapy duration, presence of fixed/matted nodes, type or number of mapping agents, it was found that the likelihood of a FNR in SLN finding significantly and independently decreased to 9.1% when at least 3 sentinel lymph nodes were examined. Another analysis of Z1071 indicated that implementing a strategy where only patients with normal axillary ultrasonography (USG) undergo SLNB reduced the FNR in patients with ≥ 2 sentinel lymph nodes removed from 12.6 to 9.8% [23]. Therefore, in our study, we included only those patients whose axillary lymph nodes showed a complete response to neoadjuvant chemotherapy according to both clinical and imaging methods, which included USG and ^18^FDG PET/CT. Additionally, we ensured that at least 3 sentinel lymph nodes were removed to maximize the reduction of the FNR of SLNB.

A phase III noninferiority study called “Tailored axillary surgery with or without axillary lymph node dissection followed by radiotherapy in patients with clinically node-positive breast cancer” (TAXIS) was designed to investigate the necessity of ALND for clinically positive nodal breast cancer in the adjuvant setting or in cases of incomplete response to neoadjuvant therapy [24]. The TAXIS trial assessed the effectiveness of tailored axillary surgery (TAS), a novel technique for selectively removing positive lymph nodes. The initial results indicated that patients in the radiotherapy-only arm were significantly understaged, as 70% of patients in the ALND arm had additional nodal disease detected during ALND, 37% of whom had stage pN2 disease [25]. Although disease-free survival data from the TAXIS study have not yet been published, it appears that patients with clinical node-positive status after neoadjuvant therapy cannot be reliably differentiated between the pN1 and pN2 stages in the SLNB/TAS arm. Therefore, the adoption of the SLNB + TAS method for patients with clinical node-positive status after neoadjuvant therapy may be challenging until it is demonstrated that there is no difference in long-term survival. Ongoing and future studies suggest that radiotherapy will play a larger role in cases with positive axilla involvement. When axilla positivity is present, there is a growing doubt about the necessity of surgery, with a preference to minimize axillary surgical procedures [26–28]. However, for patients who are clinically node negative after neoadjuvant chemotherapy, as in our present study, the SLNB results may accurately reflect the true nodal stage without the need for ALND. Currently, axillary dissection remains the standard of care for patients with residual lymph node disease confirmed by sentinel lymph node biopsy (SLNB) after neoadjuvant chemotherapy and a clinically complete response until the results of the Alliance A011202 trial are reported [5]. Thus, in our study, we identified patients who did not require ALND until long-term survival data became available.

This study has several limitations, primarily due to its retrospective design and the relatively small number of patients in the SLNB 1 + group, especially within the her-2-positive and triple-negative subgroups. Additionally, not all patients had their pathological lymph nodes marked before neoadjuvant therapy. Despite these limitations, it appears that the number of positive SLNBs and other tumor-related parameters, as well as luminal subtypes, can serve as predictors of the true axillary lymph node stage. Therefore, the patient group with only the SLNB 1 + subgroup after NAC should be analyzed separately from those with a higher count of SLNB-positive findings, and the omission of ALND should be considered for this subgroup.

## Conclusion

We may predict additional positive nodes in ALND accordingly the number of positive nodes in SLNB, tumor-related factors, and treatment response.In this study we found that in the case of SLNB 1 + positivity, it was observed that all patients in the HER-2-positive and triple-negative patient groups had no additional nodes positive at ALND. Her2-positive and triple-negative diseases were more likely to be associated with no additional nodes being positive at ALND, even in the setting of SLNB1+. Due to these results, we thought that ALND could be omitted if SLNB 1 + in Her2-positive and triple-negative subgroups who were converted to clinically node-negative after NAT. Our suggestion at this stage is to carry out extensive studies with a greater number of patients to support our results. Future studies may revise the current guidelines regarding the necessity of ALND based on SLNB results in breast cancer patients whose clinical axilla turns negative after neoadjuvant chemotherapy.

### Electronic supplementary material

Below is the link to the electronic supplementary material.

Supplementary Material 1

## Data Availability

Availability of data and materialsThe datasets used and/or analyzed during the current study available from the corresponding author on reasonable request.
