# Supplementary material for: Prediction of the number of positive axillary lymph nodes according to sentinel lymph node involvement and biological subtypes in patients receiving neoadjuvant chemotherapy
Source: BMC Surg. 2024 Jul 19;24:213. doi: 10.1186/s12893-024-02500-5 (PMC11264838; doi:10.1186/s12893-024-02500-5)
Supplement: Supplementary file 1 — Supplementary Material 1 [file 12893_2024_2500_MOESM1_ESM.pdf]

GAZİANTEP ÜNİVERSİTESİ KLİNİK ARAŞTIRMALAR ETİK KURULU KARAR FORMU

ARAŞTIRMANIN AÇIK ADI	Başlangıçta Klinik Olarak Aksilla Pozitif Olup, Neoadjuvant Kemoterapi Sonrasında Klinik ve Radyolojik Olarak Aksilla Negatife Dönüşen Meme Kanserli Hastaların Sentinel Lenf Nodu Biyopsisiyle Değerlendirilmesi ve Gereğinden Fazla Yapılan (Overtreatment) Aksiller Diseksiyonların Ortaya Konulması
VARSA ARAŞTIRMANIN PROTOKOL KODU	228

ETİK KURUL BİLGİLERİ		
	ETİK KURULUN ADI	Gaziantep Üniversitesi Klinik Araştırmalar Etik Kurulu
	AÇIK ADRESİ:	Gaziantep Üniversitesi Hayvan Deneyleri Araştırma Merkezi Binası (GAÜNDAM) Klinik Araştırmalar Etik Kurulu 27310 Şehitkamil/Gaziantep
	TELEFON	0342 360 12 00-Dahili 4800
	FAKS	-
	E-POSTA	etikkurul@gantep.edu.tr

BAŞVURU BİLGİLERİ					
	KOORDİNATÖR/SORUMLU ARAŞTIRMACI UNVANI/ADI/SOYADI	Dr. Öğr. Üyesi Latif YILMAZ			
	KOORDİNATÖR/SORUMLU ARAŞTIRMACININ UZMANLIK ALANI	Genel Cerrahi			
	KOORDİNATÖR/SORUMLU ARAŞTIRMACININ BULUNDUĞU MERKEZ	Gaziantep Üniversitesi Tıp Fakültesi			
	VARSA İDARİ SORUMLU UNVANI/ADI/SOYADI				
	DESTEKLEYİCİ				
	PROJE YÜRÜTÜCÜSÜ UNVANI/ADI/SOYADI (TÜBİTAK vb. gibi kaynaklardan destek alanlar için)				
	DESTEKLEYİCİNİN YASAL TEMSİLCİSİ				
	ARAŞTIRMANIN FAZI VE TÜRÜ	FAZ 1	☐		
		FAZ 2	☐		
		FAZ 3	☐		
		FAZ 4	☐		
		Gözlemsel ilaç çalışması	☐		
		Tıbbi cihaz klinik araştırması	☐		
		İn vitro tıbbi tanı cihazları ile yapılan performans değerlendirme çalışmaları	☐		
		İlaç dışı klinik araştırma	☒		
		Diğer ise belirtiniz :			
	ARAŞTIRMAYA KATILAN MERKEZLER	TEK MERKEZ ☒	ÇOK MERKEZLİ ☐	ULUSAL ☒	ULUSLARARASI ☐

DEĞERLENDİRİLEN BELGELER	Belge Adı	Tarihi	Versiyon Numarası	Dili		
	ARAŞTIRMA PROTOKOLÜ			Türkçe ☐	İngilizce ☐	Diğer ☐
	BİLGİLENDİRİLMİŞ GÖNÜLLÜ OLUR FORMU			Türkçe ☐	İngilizce ☐	Diğer ☐
	OLGU RAPOR FORMU			Türkçe ☐	İngilizce ☐	Diğer ☐
	ARAŞTIRMA BROŞÜRÜ			Türkçe ☐	İngilizce ☐	Diğer ☐

DEĞERLENDİRİLEN DİĞER BELGELER	Belge Adı		Açıklama
	SİGORTA	☐	
	ARAŞTIRMA BÜTÇESİ	☐	
	BİYOLOJİK MATERYEL TRANSFER FORMU	☐	
	İLAN	☐	
	YILLIK BİLDİRİM	☐	
	SONUÇ RAPORU	☐	
	GÜVENLİLİK BİLDİRİMLERİ	☐	
	DİĞER:	☐	

Etik Kurul Başkanının
Unvanı/Adı/Soyadı: Prof.Dr.Yasemin ZER

Not: Etik kurul başkanı, imzasının yer almadığı her sayfaya imza atmalıdır.

GAZİANTEP ÜNİVERSİTESİ KLİNİK ARAŞTIRMALAR ETİK KURULU KARAR FORMU

ARAŞTIRMANIN AÇIK ADI	Başlangıçta Klinik Olarak Aksilla Pozitif Olup, Neoadjuvant Kemoterapi Sonrasında Klinik ve Radyolojik Olarak Aksilla Negatife Dönüşen Meme Kanserli Hastaların Sentinel Lenf Nodu Biyopsisiyle Değerlendirilmesi ve Gereğinden Fazla Yapılan (Overtreatment) Aksiller Diseksiyonların Ortaya Konulması
VARSA ARAŞTIRMANIN PROTOKOL KODU	228

KARAR BİLGİLERİ	**Karar No:2023/228**	**Tarih: 12.07.2023**
	Sorumlu Araştırmacısı Dr. Öğr. Üyesi Latif YILMAZ olan **"Başlangıçta Klinik Olarak Aksilla Pozitif Olup, Neoadjuvant Kemoterapi Sonrasında Klinik ve Radyolojik Olarak Aksilla Negatife Dönüşen Meme Kanserli Hastaların Sentinel Lenf Nodu Biyopsisiyle Değerlendirilmesi ve Gereğinden Fazla Yapılan (Overtreatment) Aksiller Diseksiyonların Ortaya Konulması"** başlıklı proje öneri dosyası ile ilgili belgeler incelenmiş olup, etik ve bilimsel sakınca bulunmadığına toplantıya katılan etik kurul üye tam sayısının salt çoğunluğu ile karar verilmiştir.<br>İlaç ve Biyolojik Ürünlerin Klinik Araştırmaları Hakkında Yönetmelik kapsamında yer alan araştırmalar/çalışmalar için Türkiye İlaç ve Tıbbi Cihaz Kurumu'ndan izin alınması gerekmektedir.	

**KLİNİK ARAŞTIRMALAR ETİK KURULU**	
**ETİK KURULUN ÇALIŞMA ESASI**	İlaç ve Biyolojik Ürünlerin Klinik Araştırmaları Hakkında Yönetmelik<br>İyi Klinik Uygulamaları Kılavuzu
**BAŞKANIN UNVANI / ADI / SOYADI:**	Prof.Dr.Yasemin ZER

**Unvanı/Adı/Soyadı**	**Uzmanlık Alanı**	**Kurumu**	**Cinsiyet**		**Araştırma ile ilişki**		**Katılım ***		**İmza**
Prof.Dr.Yasemin ZER	Tıbbi Mikrobiyoloji	Gaziantep Üniversitesi Tıp Fakültesi	E ☐	K ☒	E ☐	H ☒	E ☐	H ☐	
Prof.Dr.Muradiye NACAK	Tıbbi Farmakoloji	Gaziantep Üniversitesi Tıp Fakültesi	E ☐	K ☒	E ☐	H ☒	E ☐	H ☐	
Prof.Dr.Osman BAŞPINAR	Çocuk Kardiyoloji	Gaziantep Üniversitesi Tıp Fakültesi	E ☒	K ☐	E ☐	H ☒	E ☐	H ☐	
Prof. Dr. Maruf ŞANLI	Göğüs Cerrahi	Gaziantep Üniversitesi Tıp Fakültesi	E ☒	K ☐	E ☐	H ☒	E ☐	H ☐	
Prof.Dr.Suzan TABUR	Endokrinoloji ve Metabolizma	Gaziantep Üniversitesi Tıp Fakültesi	E ☐	K ☒	E ☐	H ☒	E ☐	H ☐	
Doç.Dr.Beltinge DEMİRCİOĞLU KILIÇ	Çocuk Nefroloji	Gaziantep Üniversitesi Tıp Fakültesi	E ☐	K ☒	E ☐	H ☒	E ☐	H ☐	
Doç.Dr.İlkay DOĞAN	Biyoistatistik	Gaziantep Üniversitesi Tıp Fakültesi	E ☒	K ☐	E ☐	H ☒	E ☐	H ☐	İZİNLİ
Doç.Dr.Nurgül ÖZDEMİR	Psikiyatri Hemşireliği	Gaziantep Üniversitesi Sağlık Bilimleri Fakültesi	E ☐	K ☒	E ☐	H ☒	E ☐	H ☐	
Doç.Dr. İlhan BAHŞİ	Anatomi	Gaziantep Üniversitesi Tıp Fakültesi	E ☒	K ☐	E ☐	H ☒	E ☐	H ☐	
Doç.Dr.Fatih SARI	Protetik Diş Tedavisi	Gaziantep Üniversitesi Diş Hekimliği Fakültesi	E ☒	K ☐	E ☐	H ☒	E ☐	H ☐	İZİNLİ
Dr.Öğr.Üyesi Nadide Özkul DOĞRU	Fizyoloji	Gaziantep Üniversitesi Tıp Fakültesi	E ☐	K ☒	E ☐	H ☒	E ☐	H ☐	
Uzm.Dr.Hüseyin GÜRBÜZ	Acil Tıp	Dr.Ersin Arslan EAH	E ☒	K ☐	E ☐	H ☒	E ☐	H ☐	
Emine Aybuken YILDIRIM	Hukukçu	Gaziantep Barosu	E ☐	K ☒	E ☐	H ☒	E ☐	H ☐	
Zeki EKİNCİ	Sağlık Meslek Mensubu Olmayan Üye	Şahinbey Belediyesi	E ☒	K ☐	E ☐	H ☒	E ☐	H ☐	

*Toplantıda Bulunma

Etik Kurul Başkanının
Unvanı/Adı/Soyadı: Prof.Dr.Yasemin ZER

Not: Etik kurul başkanı, imzasının yer almadığı her sayfaya imza atmalıdır.
